# Rifabutin Suppresses Inducible Clarithromycin Resistance in *Mycobacterium abscessus* by Blocking Induction of *whiB7* and *erm41*

**DOI:** 10.3390/antibiotics9020072

**Published:** 2020-02-10

**Authors:** Dinah Binte Aziz, Mei Lin Go, Thomas Dick

**Affiliations:** 1 Department of Pharmacy, Faculty of Science, National University of Singapore, 18 Science Drive 4, Singapore 117543, Singapore; a0084914@u.nus.edu (D.B.A.); meilin.go@nus.edu.sg (M.L.G.); 2 Department of Medical Sciences, Hackensack Meridian School of Medicine at Seton Hall University, 340 Kingsland Street, Nutley, NJ 07110, USA; 3 Center for Discovery and Innovation, Hackensack Meridian Health, 340 Kingsland Street Building 102, Nutley, NJ 07110, USA

**Keywords:** synergy, non-tuberculous mycobacteria, NTM, rifabutin, clarithromycin, *whiB7*, *erm41*

## Abstract

Clarithromycin (CLR) is the corner stone in regimens for the treatment of lung disease caused by *Mycobacterium abscessus (Mab)*. However, many strains harbor the CLR-inducible CLR resistance gene *erm41,* encoding a ribosome methylase. Induction of *erm41* is mediated by the transcription factor *whiB7*. We hypothesized that an inhibitor of RNA synthesis should be able to block the *whiB7–erm41* induction response to CLR exposure and thus suppress CLR resistance. Recently, we discovered that the rifampicin analog rifabutin (RFB) shows attractive potency against *Mab*. To determine whether RFB-CLR combinations are synergistic, a checkerboard analysis against a collection of *erm41* positive and negative *Mab* strains was carried out. This revealed synergy of the two drugs for *erm41* positive but not for *erm41* negative strains. To determine whether RFB’s potentiation effect was due to inhibition of the transcriptional induction of the *whiB*7–*erm41* resistance system, we measured the effect of CLR alone and in combination with RFB on *whiB7* and *erm41* mRNA levels. CLR alone strongly induced *whiB7* and *erm41* expression as expected. The synergistic, growth-inhibiting combination of RFB with CLR blocked induction of both genes. These results suggest that RFB suppresses inducible CLR resistance by preventing induction of *whiB*7 and *erm41* expression.

## 1. Introduction

*Mycobacterium abscessus* (*Mab*) causes difficult-to-cure lung disease. Current multidrug-regimens typically include clarithromycin (CLR) (or its analog azithromycin), amikacin, imipenem and tigecycline [1,2,3]. Rifampicin, a key drug used for the treatment of *Mycobacterium tuberculosis,* shows poor activity against *Mab* and is not used clinically. CLR is the cornerstone of *Mab* treatment and resistance against CLR is associated with poor outcomes [4,5]. Many *Mab* strains are intrinsically resistant against CLR. Intrinsic resistance is mediated by Erm41 [6]. Erm41 is a methylase that modifies the binding site of CLR on the 23S ribosomal RNA. Intrinsic CLR resistance is inducible. Exposure to subinhibitory concentrations of CLR induces transcription of *whiB7* encoding a transcriptional activator, which in turn induces transcription of *erm41* [6,7,8,9].

*Mab* represents a complex of three subspecies, *Mab* subsp. *abscessus*, *Mab* subsp. *bolletii* and *Mab* subsp. *massiliense* [10]. Interestingly, all *Mab* strains carry *erm41* sequences, but only a subset harbors functional *erm41* alleles, i.e., alleles that confer phenotypic CLR resistance (‘*erm41* positive’ strains). The others (‘*erm41* negative’) are CLR susceptible strains that carry deletions or a T to C polymorphism at position 28 of the coding sequence of the gene, resulting in loss of Erm41 activity [11,12,13,14]. 

New drugs that address CLR resistant *Mab* infections, are urgently needed [15]. In a repurposing project, we recently screened a library of approved drugs and found attractive in vitro activity in the rifampicin analog rifabutin (RFB) [16]. Subsequently, we demonstrated activity of RFB in a mouse model of *Mab* infection [17], and a first report on the beneficial use of the drug in patients suffering from disseminated Mab disease was published [5]. This suggests that RFB is a repurposing candidate for the treatment of *Mab* lung infections. Based on the finding that inducible CLR resistance appears to be under transcriptional control and that exposure to CLR increases the mRNA levels of both *whiB7* and *erm41*, we hypothesized that treatment of *Mab* cultures with RFB should suppress inducible resistance. The RNA polymerase inhibitor should block transcriptional induction of the *whiB7-erm41* resistance system and thus hold the genotypically CLR resistant *Mab* in a phenotypically CLR susceptible state. This hypothesis is supported by recent synergy studies that suggest that RFB can potentiate the activity of CLR [18,19]. If confirmed, this would suggest that intrinsically CLR-resistant *Mab* disease due to *erm41* could become treatable with CLR by adding RFB. Here, we tested this hypothesis by determining the effect of RFB on CLR activity in *erm41* positive and negative strains in vitro, and by determining the effect of RFB on transcript levels of *whiB7* and *erm41*. 

## 2. Results

**The RFB-CLR combination is synergistic for *erm41* positive and indifferent for *erm41* negative *M. abscessus*.** If RFB suppresses inducible CLR resistance, RFB should potentiate the effect of CLR. This potentiation effect should result in synergy of the two drugs. The synergistic effect of CLR-RFB should be specific for *erm41* positive *Mab* strains and should not be observed for *erm41* negative strains. To test these predictions, we carried out a checkerboard assay to determine drug–drug potency interactions. The assay was carried out with a collection of reference strains and clinical isolates covering all three subspecies and containing both *erm41* positive and negative strains. The CLR-RFB combination was indeed synergistic against all *erm41* positive strains (Table 1). This suggests that RFB potentiates the effect of CLR in this genetic background, as expected if RFB suppresses the expression of CLR resistance and thus increases the susceptibility to the antibiotic. Furthermore, as predicted, the RFB-CLR combination was indifferent to all *erm41* negative *Mab* strains, demonstrating that RFB required the presence of inducible *erm41* to potentiate the activity of CLR. Thus, RFB potentiates CLR in an *erm41* dependent manner. It is interesting to note that the outcomes of the drug–drug interaction studies were solely dependent on the *erm41* status (type of *erm41* sequevar) and not influenced by the type of subspecies. This is illustrated by the difference in outcome between the two *Mab* subspec. *abscessus* isolates Bamboo (*erm41* negative) and *Mab* ATCC19977 (*erm41* positive), and the two *Mab* subspecies *bolletii* isolates M506 (*erm41* negative) and CCUG 50184-T (*erm41* positive). Independent of the subspecies, RFB-CLR synergy was observed for *erm41* positive members of the respective subspecies (Table 1). In summary, the checkerboard analyses showed that the RFB-CLR combination is synergistic for *erm41* positive and indifferent for *erm41* negative *Mab* strains. This shows that the potentiation effect of RFB on CLR is *erm41* dependent. Thus, co-treatment of *Mab* strains displaying inducible CLR resistance with RFB appears to restore activity of CLR.

**Figure 1:**
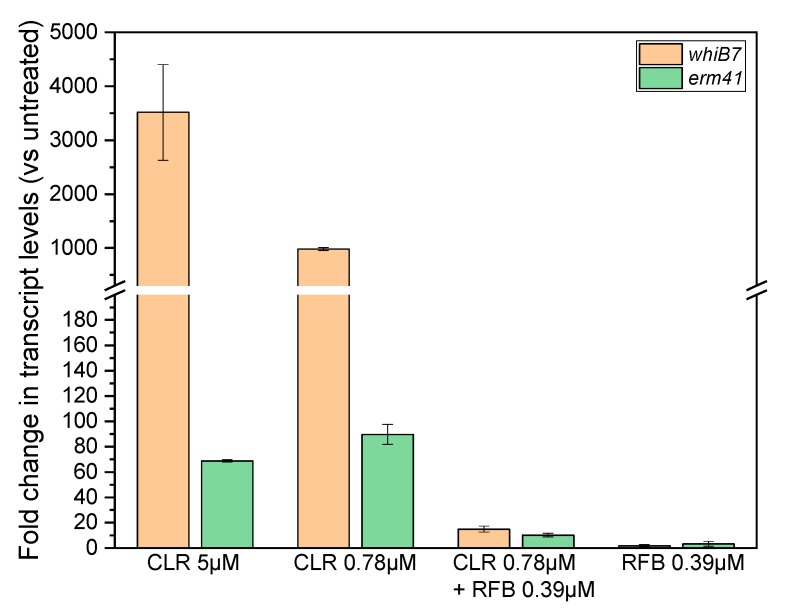
Effect of treatment of *M. abscessus* with clarithromycin and/or rifabutin on *whiB7* and *erm41* mRNA levels. *M. abscessus* ATCC19977 cultures were exposed for 30 min to clarithromycin (CLR), rifabutin (RFB) or a combination of both antibiotics and relative transcript levels were determined. Fold changes compared to untreated cultures are shown. CLR 5 µM: MIC of CLR. CLR 0.78 µM: subinhibitory CLR concentration used in the synergistic combination. CLR 0.78 µM + RFB 0.39 µM: synergistic, growth inhibitory combination. RFB 0.39 µM: subinhibitory RFB concentration used in the synergistic combination. Transcript levels were measured by qRT-PCR and normalized to *rpsA* as described [6]. Shown are mean values with standard deviations from two independent experiments. 0.78 µM CLR corresponds to 1 mg/L; 0.39 µM RFB corresponds to 0.46 mg/L.

**Treatment of *erm41* positive *M. abscessus* with RFB-CLR combination at synergistic, growth inhibiting concentrations suppresses induction of *whiB7* and *erm41* expression.** Treatment of *erm41* positive *Mab* with CLR induces transcription of *whiB7* and *erm41* [6,7]. The checkerboard analysis revealed that growth of the *erm41* positive *Mab* ATCC19977 is inhibited by a combination of CLR and RFB at 0.78 and 0.39 µM, respectively (Table 1 and Table S1). This compares to the MIC of the single drugs of 5 µM (CLR) and 3 µM (RFB). If this synergistic effect of the CLR-RFB combination is due to blocking induction of transcription of *whiB7* and *erm41*, the mRNA levels of the two genes should not increase in cultures treated with the synergistic, growth inhibiting CLR-RFB combinations. To determine the effect of drug treatment on the mRNA levels of the two genes, cultures were treated for 30 min and quantitative reverse transcription PCR (qRT-PCR) was carried out. First, we confirmed that CLR alone at MIC (5 µM) or a subinhibitory concentration of 0.78 µM causes an increase of *whiB7* and *erm41* mRNA levels as reported previously (Figure 1). Then we determined the effect of the synergistic, growth inhibitory RFB-CLR combination. Adding 0.39 µM RFB to 0.78 µM CLR suppressed the increase of both *whiB7* and *erm41* transcript levels (Figure 1). Taken together, these results confirm that CLR treatment alone induces transcription of *whiB7* and *erm41* and show that the addition of RFB blocks this induction. These results suggest that the observed potentiation effect of RFB on CLR is due to the inhibition of induction of the *whiB7-erm41* resistance system.

## 3. Discussion

Repurposing and repositioning of antibacterials used in the clinic is an attractive alternative to de novo drug discovery [15]. To identify clinically used medications active against *Mab*, we recently screened a library of approved drugs and identified the rifamycin RFB to be active against *Mab* in vitro [16]. This was surprising because rifampicin, the corner stone drug for *M. tuberculosis* treatment, shows poor activity against *Mab* and is therefore not used clinically. The reason for the difference in potency of the two rifamycins against *Mab* remains to be determined but appears to involve bacterial metabolism [20,21]. Thus, RFB, being an approved oral drug, should be considered for repurposing as an add-on for the treatment of *Mab* lung disease. This notion is supported by our recent finding, that RFB, in contrast to rifampicin, also showed efficacy in vivo in a mouse model for *Mab* infection [17]. The first report suggesting that RFB may be beneficial for the treatment of patients suffering from disseminated *Mab* disease is encouraging [5].

CLR is a key component in current treatments of *Mab* lung disease. However inducible CLR resistance conferred by the *whiB7-erm41* resistance system is widespread among clinical isolates [11,12]. The induction of *whiB7-erm41* mediated CLR resistance appears to be regulated at the transcriptional level [6,7,22,23]. Thus, we asked here whether the RNA synthesis inhibitor RFB may suppress inducible CLR resistance. We show that RFB indeed potentiated the activity of CLR in *erm41* positive strains (Table 1). This potentiation effect of RFB on CLR correlated with a suppression of the transcriptional induction of the *whiB7* and *erm41* genes (Figure 1). These results show that RFB suppresses phenotypic expression of CLR resistance, i.e., RFB treatment appears to keep *Mab* in a CLR susceptible state. Furthermore, our results provide a mechanistic explanation for this effect, namely that RFB suppresses transcriptional activation of *whiB7*-*erm41*.

The presented work may have clinical implications. It suggests that RFB may not only be useful as an add-on to the regimens used for the treatment of *Mab* lung disease in general as suggested earlier [16], but may be particularly useful for disease caused by *erm*41 positive *Mab* as the rifamycin appears to restore CLR susceptibility of the genotypically resistant bacteria (Ganapathy et al., 2019).

Our study has several limitations. One limitation of the current work is that we tested only a small collection (*n* = 16) of *Mab* strains in our RFB-CLR drug–drug potency interaction analysis. However, the conclusion that RFB potentiates CLR in an *erm41* dependent manner is supported by previous studies, which showed similar trends [18,19]. A second limitation is, that we did not confirm the proposed mechanism by generating and analyzing targeted knock-outs in *whiB7* and *erm41*. Finally, all the analyses were carried out in vitro and the effects need to be demonstrated in an animal model of *Mab* infection.

## 4. Conclusions

We have shown that co-treatment of *erm41* positive *Mab* with RFB and CLR in vitro suppresses induction of CLR resistance. This effect appears to be due to inhibition of CLR mediated induction of the *whiB7-erm41* system. This suggest that RFB-CLR treatment may be beneficial for patients infected with *erm41* positive *Mab*.

## 5. Materials and Methods 

**Compounds.** Clarithromycin was from Sigma-Aldrich and dissolved in acetone. Rifabutin was from SelleckChem and dissolved in 90% dimethyl sulfoxide (DMSO). 

**Bacterial strains and culture media.** For the checkerboard titration assay, both clinical isolates as well as *Mycobacterium abscessus* reference strains were used. The reference strains were obtained from the American Type Culture Collection (ATCC) and the Culture Collection University of Goteborg (CCUG) and comprised *Mycobacterium abscessus* subsp. *abscessus* (ATCC 19977), *Mycobacterium abscessus* subsp. *bolletii* (CCUG 50184-T) and *Mycobacterium abscessus* subsp. *massiliense* (CCUG 48898-T). The clinical isolates were obtained from the strain collection of the clinical microbiology laboratory at the National University Hospital, Singapore. The clinical strains were characterized previously [16]. For the measurement of the RNA levels by quantitative reverse transcription PCR (qRT-PCR), *M. abscessus* subsp. *abscessus* (ATCC 19977) was used. Liquid cultures were grown in standard mycobacterium medium, Middlebrook 7H9 broth (BD Difco) supplemented with 0.5% albumin, 0.2% glucose, 0.085% sodium chloride, 0.0003% catalase, 0.2% glycerol and 0.05% Tween 80. 

**Checkerboard titration assay.** This assay was carried out in 96-well microtiter plates as previously described using optical density to assess growth [24]. Eight concentrations of CLR were tested. For the *erm41* negative strains, the concentration range was 0–1.25 µM. For the *erm41* positive strains, the concentration range was 0–12.5 µM. The CLR concentrations were tested against 11 concentrations of rifabutin from 0–25 µM. Concentrations used were two-fold serial dilutions from the highest concentration. The fractional inhibitory concentration index (FICI) was used to analyze the results from the checkerboard assay. FICI was calculated by using the concentrations at which at least 90% inhibition of the culture in the well as compared to the drug free culture was observed. It was computed as FICI = [(concentration of drug A in combination/concentration of drug A when used alone) + (concentration of drug B in combination/concentration of drug B when used alone) [25]. Synergy was defined as FICI ≤ 0.5, indifference was defined as 0.5 < FICI ≤ 4, and antagonism was defined as FICI > 4 [25].

**RNA extraction, quantitative reverse transcription PCR (qRT-PCR) analysis.***M. abscessus* subsp. *abscessus* (ATCC 19977) mid-log phase cultures were diluted to OD_600_ = 0.1 and exposed to antibiotics for 30 minutes. Cells were concentrated to OD_600_ = 0.5 and then lysed with Trizol (Ambion) and a mechanical disruption step using lysing matrix B in the FastPrep-24 5G (MP Biomedical). RNA was extracted using the PureLink RNA Mini Kit (Ambion), with on-column DNase (Ambion) treatment. The amount of RNA used was 50 ng/µL for all conditions. Random primers (Promega) and Superscript III reverse transcriptase (Invitrogen) were used for cDNA synthesis in the presence of RNase OUT (Invitrogen). Conditions for cDNA synthesis were: 50 °C for 30 min, followed by 70 °C for 15 min. cDNA was then subjected to qPCR using FastStart Essential DNA Green Master (Roche) along with previously described primers; *whiB7* (5’-CCTGTGGTTCGCGGAAA-3’/5’-CCCTGCTCAAGAATCTCACC-3’) and *erm41* (5’-CGAGCCCGCCCTACCAAGTCAC-3’/5’-CCGGCCCGTAGCGTCCAATG-3’) [6,7]. Results were normalized to *rpsA* using the previously described primer set; *rpsA* (5’-CAAGTAGCCGTCAACGACA-3’/5’-GACACCTTCGGTCTTGTAAC-3’) [6]. A LightCycler 96 (Roche) was used for qPCR. Cycling conditions were: 95 °C for 10 min, followed by 45 cycles of 95 °C for 10 s, 60 °C for 10 s and 72 °C for 10 s. Results are presented as expression fold change of treated sample compared to untreated. 

## Figures and Tables

**Table 1:** Checkerboard assay results for the combination of clarithromycin and rifabutin against three reference strains and a collection of clinical isolates of *M. abscessus*.

Isolate Code	*M. Abscessus* Subspecies	*Erm41* Sequevar	*Erm41* Status	CLR Susceptibility	CLR + RFB	FICI
ATCC 19977	*abscessus*	T28	Functional	Resistant	S	0.26
Bamboo	*abscessus*	C28	Non-functional	Sensitive	I	0.56
M9	*abscessus*	T28	Functional	Resistant	S	0.39
M199	*abscessus*	T28	Functional	Resistant	S	0.39
M337	*abscessus*	T28	Functional	Resistant	S	0.39
M421	*abscessus*	T28	Functional	Resistant	S	0.49
M422	*abscessus*	T28	Functional	Resistant	S	0.39
CCUG 50184-T	*bolletii*	T28	Functional	Resistant	S	0.32
M232	*bolletii*	T28	Functional	Resistant	S	0.21
M506	*bolletii*	C28	Non-functional	Sensitive	I	0.77
CCUG 48898-T	*massiliense*	deletion	Non-functional	Sensitive	I	0.77
M111	*massiliense*	deletion	Non-functional	Sensitive	I	0.78
M353	*massiliense*	deletion	Non-functional	Sensitive	I	0.65
M357	*massiliense*	deletion	Non-functional	Sensitive	I	1.05
M414	*massiliense*	deletion	Non-functional	Sensitive	I	0.86
M444	*massiliense*	deletion	Non-functional	Sensitive	I	0.85
M505	*massiliense*	deletion	Non-functional	Sensitive	I	1.17

Fractional inhibitory concentration index (FICI) values are shown as a measure of synergy. Synergy (S) is defined as FICI ≤ 0.5, indifference (I) is defined as 0.5 < FICI ≤ 4, and antagonism (A) is defined as FICI > 4. *erm41* status (functional = *erm41* positive; non-functional = *erm41* negative) and phenotypic CLR susceptibility of the strains were described previously [16].

## Supplementary Materials

The following are available online at https://www.mdpi.com/2079-6382/9/2/72/s1, Table S1: Detailed MIC_90_ values (in µM) of checkerboard assay results for the combination of clarithromycin and rifabutin against three reference strains and a collection of clinical isolates of *M. abscessus.*
