# Supplementary material for: Rifabutin Suppresses Inducible Clarithromycin Resistance in Mycobacterium abscessus by Blocking Induction of whiB7 and erm41
Source: Antibiotics (Basel). 2020 Feb 10;9(2):72. doi: 10.3390/antibiotics9020072 (PMC7168051; doi:10.3390/antibiotics9020072)
Supplement: Supplementary file 1 [file antibiotics-09-00072-s001.pdf]

Table S1: Detailed MIC_{90} values (in μM) of checkerboard assay results for the combination of Clarithromycin and Rifabutin against three reference strains and a collection of clinical isolates of *M. abscessus.*

			Alone		In combination	
**Isolate code**	**CLR + RFB**	**FICI**	**CLR (μM)**	**RFB (μM)**	**CLR (μM)**	**RFB (μM)**
ATCC 19977	S	0.26	6	3	0.78	0.39
Bamboo	I	0.56	0.5	1.5	0.15	0.39
M9	S	0.39	3	3	0.78	0.39
M199	S	0.39	12	3	1.56	0.78
M337	S	0.39	12	3	1.56	0.78
M421	S	0.49	2	2	0.78	0.19
M422	S	0.39	2	2	0.39	0.39
CCUG 50184-T	S	0.32	3	3	0.19	0.78
M232	S	0.21	10	2	0.19	0.39
M506	I	0.77	0.4	2	0.15	0.78
CCUG 48898-T	I	0.77	0.2	0.5	0.078	0.19
M111	I	0.78	0.4	3	0.31	0.04
M353	I	0.65	0.4	3	0.156	0.78
M357	I	1.05	0.6	4	0.625	0.04
M414	I	0.86	0.5	4	0.039	3.125
M444	I	0.85	0.6	4	0.039	3.125
M505	I	1.17	0.4	4	0.312	1.56

Fractional Inhibitory Concentration Index (FICI) values are shown as a measure of synergy. Synergy (S) is defined as FICI ≤0.5, Indifference (I) is defined as 0.5<FICI≤4, and Antagonism (A) is defined as FICI >4. *erm41* status (functional = *erm41* positive; non-functional = *erm41* negative) and phenotypic CLR susceptibility of the strains were described previously.
